# Supplementary material for: Development of a Multienzyme Isothermal Rapid-Amplification Lateral Flow Assay for On-Site Identification of the Japanese Eel (Anguilla japonica)
Source: Foods. 2025 Sep 4;14(17):3100. doi: 10.3390/foods14173100 (PMC12428543; doi:10.3390/foods14173100)
Supplement: Supplementary file 1 [file foods-14-03100-s001.zip › foods-3846833-supplementary.pdf]

Table S1. Reference mitochondrial genome sequences of *Anguilla* species retrieved from the NCBI GenBank.

Species	Accession number	Number of data
Anguilla japonica	AB038556.2, NC002707.2, CM002536.1, KJ948424.1, KT355033.1, MH050933.1	6
Anguilla anguilla	AP007233.1, NC006531.1, KJ564218.1, KJ564219.1, KJ564220.1, KJ564221.1, KJ564222.1, KJ564223.1, KJ564224.1, KJ564225.1, KJ564226.1, KJ564227.1, KJ564228.1, KJ564229.1, KJ564230.1, KJ564231.1, KJ564232.1, KJ564233.1, KJ564234.1, KJ564235.1, KJ564236.1, KJ564237.1, KJ564238.1, KJ564239.1, KJ564240.1, KJ564241.1, KJ564242.1, KJ564243.1, KJ564244.1, KJ564245.1, KJ564246.1, KJ564247.1, KJ564248.1, KJ564249.1, KJ564250.1, KJ564251.1, KJ564252.1, KJ564253.1, KJ564254.1, KJ564255.1, KJ564256.1, KJ564257.1, KJ564258.1, KJ564259.1, KJ564260.1, KJ564261.1, KJ564262.1, KJ564263.1, KJ564264.1, KJ564265.1, KJ564266.1, KJ564267.1, KJ564268.1, KJ564269.1, KJ564270.1, MT410930.1	56
Anguilla rostrata	AP007249.2, NC006547.2, KJ564170, KJ564171, KJ564172, KJ564173, KJ564174, KJ564175, KJ564176, KJ564177.1, KJ564178.1, KJ564179.1, KJ564180.1, KJ564181.1, KJ564182.1, KJ564183.1, KJ564184.1, KJ564185.1, KJ564186.1, KJ564187.1, KJ564188.1, KJ564189.1, KJ564190.1, KJ564191.1, KJ564192.1, KJ564193.1, KJ564194.1, KJ564195.1, KJ564196.1, KJ564197.1, KJ564198.1, KJ564199.1, KJ564200.1, KJ564201.1, KJ564202.1, KJ564203.1, KJ564204.1, KJ564205.1, KJ564206.1, KJ564207.1, KJ564208.1, KJ564209.1, KJ564210.1, KJ564211.1, KJ564212.1, KJ564213.1, KJ564214.1, KJ564215.1, KJ564216.1, KJ564217.1, KJ564271.1, CM003870.1, MT667251.1, CM031800.2	54
Anguilla bicolor pacifica	AP007237.3, NC006535.3, CM077320.2	3
Anguilla marmorata	AP007242.1, NC006540.1, OR475302.1	3
Anguilla australis	PP405427.1, NC087905.1, AP007234.1, NC006532.1, AP007235.1, NC006533.1	6
Anguilla bengalensis bengalensis	KT895265.1	1
Anguilla bengalensis labiata	NC006543.1, AP007245.1	2
Anguilla bicolor bicolor	AP007236.1, NC006534.1	2
Anguilla celebesensis	AP007239.1, NC006537.1	2
Anguilla dieffenbachii	AP007240.1, NC006538.1	2
Anguilla interioris	AP007241.1, NC006539.1	2
Anguilla luzonensis	AB469437.1, NC011575.1	2
Anguilla malgumora	AP007238.1, NC006536.1	2
Anguilla megastoma	AP007243.1, NC006541.1	2
Anguilla mossambica	AP007244.1, NC006542.1	2
Anguilla nebulosa	AP007246.1, NC006544.1	2
Anguilla obscura	AP007247.1, NC006545.1	2
Anguilla reinhardtii	AP007248.1, NC006546.1	2

Total	153
